# Direct measurement of pervasive weak repression by microRNAs and their role at the network level

**DOI:** 10.1186/s12864-018-4757-z

**Published:** 2018-05-15

**Authors:** Fuqiang Ma, Pei Lin, Qingjian Chen, Xuemei Lu, Yong E. Zhang, Chung-I Wu

**Affiliations:** 1 Key Laboratory of Genomic and Precision Medicine, Beijing Institute of Genomics, Chinese Academy of Sciences, Beijing, 100101 China; 2 University of Chinese Academy of Sciences, Beijing, 100049 China; 3 School of Life Science, Sun Yat-Sen University, Guangzhou, 510275 Guangdong China; 4 State Key Laboratory of Integrated Management of Pest Insects and Rodents & Key Laboratory of Zoological Systematics and Evolution, Institute of Zoology, Chinese Academy of Sciences, Beijing, 100101 China; 5 Department of Ecology and Evolution, University of Chicago, Chicago, IL 60637 USA; 6 Center for Excellence in Animal Evolution and Genetics, Chinese Academy of Sciences, Kunming, 650223 China

**Keywords:** mRNA degradation, MiR310s, Targets, Weak repression, Network

## Abstract

**Background:**

A gene regulatory network (GRN) comprises many weak links that are often regulated by microRNAs. Since miRNAs rarely repress their target genes by more than 30%, doubts have been expressed about the biological relevance of such weak effects. These doubts raise the possibility of under-estimation as miRNA repression is usually estimated *indirectly* from equilibrium expression levels.

**Results:**

To measure miRNA repression directly, we inhibited transcript synthesis in *Drosophila* larvae and collected time-course data on mRNA abundance, the decline of which reflects transcript degradation. The rate of target degradation in the absence of miR310s, a moderately expressed miRNA family, was found to decrease by 5 to 15%. A conventional analysis that does not remove transcript synthesis yields an estimate of 6.5%, within the range of the new estimates. These data permit further examinations of the repression mechanisms by miRNAs including seed matching types, APA (alternative polyadenylation) sites, effects of other highly-expressed miRNAs and the length of 3’UTR. Our direct measurements suggest the latter two factors have a measurable effect on decay rate.

**Conclusion:**

The direct measurement confirms pervasive weak repression by miRNAs, supporting the conclusions based on indirect assays. The confirmation suggests that this weak repression may indeed be miRNAs’ main function. In this context, we discuss the recent proposal that weak repression is “cumulatively powerful” in stabilizing GRNs.

**Electronic supplementary material:**

The online version of this article (10.1186/s12864-018-4757-z) contains supplementary material, which is available to authorized users.

## Background

In any gene regulatory network (GRN), there is a small percentage of gene-to-gene interactions whereby the regulatory gene (usually a transcription factor, TF) up- or down-regulates its target genes by several fold, often measured using ChIP-seq -type analyses [1, 2]. The phenotypic consequences and functional significance of such strong interactions have been extensively documented [3, 4]. In contrast, a different group of regulatory molecules, namely the microRNAs (miRNAs), repress hundreds of targets per miRNA weakly and broadly [5–7] in the GRNs of metazoans. Each miRNA binds mainly to the 3′ untranslated regions (3’UTR) of target transcripts that have a sequence matching the 7–8 bp seed region of the miRNA. The binding induces transcript degradation and/or translational inhibition [5, 8–10].

The broad and weak repression by miRNAs has been a central conundrum in the control of GRNs. A view that has been increasingly supported is the “few targets” hypothesis [11–14]. In this view, only the most strongly repressed targets are biologically meaningful. In some cases, a single gene (such as the *let-7* miRNA) is believed to repress a single target (*lin-41*) to yield a dramatic phenotypic consequence [12]. In contrast, Hunter et al. (2013), analyzing the same *let-7* miRNA and the same phenotype in the same species, reach the opposite conclusion [15]. They suggest that more than 20 target genes are involved in controlling the vulva phenotype. At the center of the debate is the two aspects of the same issue: First, is there a single regulator-target interaction that is strong enough to exert a phenotypic effect? Second, if not, then what might be the functions of all those weak interactions?

Liufu et al. (2017) have recently shown that each regulatory molecule (miR310s in this case) governs the same phenotype through multiple targets incoherently [16]. For example, miR310s enhances egg hatchability by repressing the *Mad* target gene but reduces hatchability via three other targets, *E2f2*, *EcR*, and *Mef2*. They conclude that the many weak repressions function to stabilize the phenotype. If the task is to correct any deviations from the norm, weak effects should suffice. Chen et al. (2017; in review but posted on BioRxiv) introduces the May-Wigner theory used in studying food web stability to the analysis of GRNs [17]. They conclude that the weak miRNA regulatory effects are cumulative. Repression each of 50 targets by 1% has a greater effect on system stability than repression of one target gene by 50% in their simulations.

At the heart of the debate, theoretical arguments, and empirical tests is the assertion that miRNAs indeed weakly repress many targets. However, this conclusion is based on indirect measurements. When an miRNA is deleted, it is generally observed that the expression of predicted target genes increases [5]. However, the increase is usually very modest. It is often less than 20% for even highly-expressed miRNAs [18, 19], and even smaller for the less expressed molecules [20]. Such weak effects have led to two different views. In one view, the pervasive weak expression is biologically real as miRNAs exert their influence through the entire RNA:RNA network. Weak effects function cumulatively at the systems level [21–24].

In contrast, as one of the conventional explanations, the weak repression could be the result of biased measurements [25–30]. It is sometimes suggested that miRNAs’ effects should be measured at the level of proteins, not mRNAs [31–33]. Several studies address this issue directly [34–37] and report that mRNA and protein measurements are reasonably well correlated. Given this correlation, it seems unlikely that weak repressions of, say, 10% at the mRNA level would be translated into strong repressions of, say, 50% at the protein level.

In this study, we further explore the possibility that miRNA effects are under-estimated. Previous estimates are affected by both mRNA synthesis and degradation. Therefore, the rate of degradation could potentially be severely under-estimated if the two processes are inter-dependent as several studies have concluded [38–40]. Here, we measure miRNA target degradation directly by turning off transcription using Actinomycin D (ActD), a widely-used chemical for transcription inhibition [41]. We took care to apply the drug only for a short duration and monitored larval viability to reduce the likelihood of side effects.

The miR310 cluster (miR310s) of *D. melanogaster* is chosen for this study because its function, especially in the context of evolution in *Drosophila*, has been analyzed in some detail [42–46]. The mid-level expression level of this cluster [47, 48] also makes it a suitable candidate for addressing the estimation of miRNA repression levels.

## Results

### I. Theory

Let *x*_*i*_*(t)* denote the mRNA concentration of a miRNA target gene at time *t*. When the system is at an equilibrium, $\frac{{\mathrm{dx}}_{\mathrm{i}}}{\mathrm{dt}}=0$ (also dx_j_/dt = 0 for all other genes *j*). Near equilibrium, we approximate transcript change by a linear system. For gene i, the system is described by the following equation (Eq.)

1 $$ {\mathrm{dx}}_{\mathrm{i}}/\mathrm{dt}={\mathrm{B}}_{\mathrm{i}}\hbox{--} {\mathrm{D}}_{\mathrm{i}}{\mathrm{x}}_{\mathrm{i}} $$

where B_i_ (= b_i_ + S_i_) is the synthesis rate and D_i_ (= d_i_ + m_i_) is the degradation rate.

Here, b_i_ is the presumably constant basal transcription rate and

2 $$ \mathrm{Si}={\sum}_{\mathrm{j}=1,\mathrm{j}\ne \mathrm{i}}^{\mathrm{N}} aijxj $$

is the aggregate effect of other genes on gene *i* with *a*_*ij*_ being the regulation strength of gene *i* by gene *j*. Further, d_i_ is the degradation rate of transcript *i* in the absence of a matching miRNA and m_i_ is the added degradation due to miRNA action. At equilibrium, X_i_ = B_i_/D_i_ where the capital letter indicates the equilibrium value. The equations can also be applied to the genetic background where the regulatory miRNA is deleted. In that background, X_i_*, B_i_* and D_i_* are substituted into Eqs. (1–2) and X_i_* = B_i_*/d_i_ (note that D_i_* = d_i_).

To quantify the repression effect of miRNAs, one would compare the two equilibrium values, X_i_ (= B_i_/D_i_) vs. X_i_* (= B_i_*/d_i_). It is usually assumed that B_i_/B_i_* ~ 1. Hence, if X_i_*/ X_i_ = 1.1, the interpretation is that D_i_/d_i_ = (d_i_ + m_i_)/d_i_ = 1.1 and m_i_ is said to increase the degradation by about 10%. In reality, the ratio of B_i_/B_i_* is nearly impossible to quantify because B_i_ and B_i_* both contain the summation term of Eq. (2), where x_j_’s and x_j_*‘s are very likely different. An incorrect assumption of B_i_/B_i_* ~ 1 could thus lead to potentially serious under-estimation of miRNAs repression activity. For example, if X_i_*/ X_i_ = 1.1 and B_i_/B_i_* = 2, then (d_i_ + m_i_)/d_i_ = 2.2. In that case, the miRNA actually increases degradation by 120% instead of 10%.

Given that the confounding factor of B_i_/B_i_* cannot be estimated, we apply an Actinomycin D (ActD) solution to inhibit transcription, making B_i_ = B_i_* = 0. Eq. (1) is reduced to

3 $$ {\mathrm{dx}}_{\mathrm{t}}/\mathrm{dt}=\hbox{-} \left(\mathrm{d}+\mathrm{m}\right)\ {\mathrm{x}}_{\mathrm{t}} $$

4 $$ {{\mathrm{dx}}_{\mathrm{t}}}^{\ast }/\mathrm{dt}=\hbox{-} \mathrm{d}\ {{\mathrm{x}}_{\mathrm{t}}}^{\ast } $$

in the wild-type (WT) and knockout (KO) background, respectively. As we only deal with gene i but at different time points, the subscript i is replaced by t. x_t_ is measured by RNA-Seq at different time points after ActD is administered as described below.

### II. Direct vs. indirect measurement of transcript decay

#### Transcription shutdown in vivo in Drosophila larvae

ActD is the standard reagent to repress both rRNA and mRNA synthesis [49, 50]. We chose the L3 stage during larval development for transcription repression because larvae appear to be able to withstand ActD treatment for 8 h without dying. When L3 larvae are immersed in an 80 μg/ml of ActD solution for 8 h, all larvae remain viable and do not appear to show obvious abnormalities. To ensure that a sufficient concentration of ActD is administered without causing undue damage to the cells, a preliminary experiment was done using a gradient of ActD concentration. As ActD affects rRNA synthesis at a much lower concentration than it affects mRNA synthesis, we expect the proper ActD concentration sufficient to disrupt mRNA production should lead to difficulties in pupation and eclosion. We chose 80 μg/ml for which the eclosion rate is still appreciable, more than 10% of the normal rate. In either the WT or miR310s-KO background, x_t_ is measured by RNA-Seq at 4 time points: 0, 2, 4, and 8 h after ActD is administered at the pre-determined concentration. A high between-replicate correlation was observed (Additional file 1: Figure S1).

On the basis of Eqs (3) and (4), we obtain

5 $$ \ln\ \left({\mathrm{x}}_{\mathrm{t}}\right)=\ln\ \left(\mathrm{B}/\mathrm{D}\right)\hbox{--} \left(\mathrm{d}+\mathrm{m}\right)\ \mathrm{t} $$

6 $$ \ln\ \left({{\mathrm{x}}_{\mathrm{t}}}^{\ast}\right)=\ln\ \left({\mathrm{B}}^{\ast }/\mathrm{d}\right)\hbox{--} \left(\mathrm{d}\right)\ \mathrm{t}. $$

**Fig. 1:**
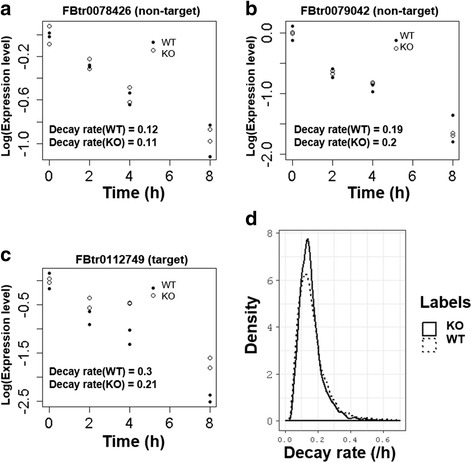
Degradation plots of (**a**-**b**) two untargeted transcripts, (**c**) one miR310s targeted transcript and (**d**) all transcripts. The expression level at 0 h is adjusted to 1. D is estimated by fitting the exponential model with log transformed normalized expression values. The slope corresponds to the decay rate

When ln (x_t_) or ln (x_t_*) is plotted against t (see Methods), the regression slope is d + m in the WT or d in the knock-out background. Plots of three representative transcripts including two miR310s non-target and one target gene are given in Fig. 1a-c. Note that the intercept in each panel is not informative because the intercept, unlike the slope, depends on the calibration of x_t_’s (see legends for detail).

The distribution of the decay rate D (= d + m) in the WT background is given in Fig. 1d. In computing the distribution, we restrict the analysis to 4500 most highly-expressed transcripts to control for the uncertainty in estimating D for weakly expressed genes. The distribution has a long tail on the right indicating that a fraction of transcripts do decay with extreme rapidity. Because the corresponding distribution of D* (=d) in the knockout background exhibits the same trend (Fig. 1d), the analysis suggests that the rapid decay is not a consequence of miRNA action. Furthermore, there exists a small fraction of transcripts that decay at an exceptionally low rate. This can be seen if we plot the distribution of half-life of all transcripts (t_1/2_ = ln2/D) as shown in Additional file 2: Figure S2. The tail to the right indicates exceptionally slow decay. The degree of kurtosis seen in the combined Fig. 1d and Additional file 2: Figure S2 could make the comparison of two distributions (in the WT vs. KO background) more difficult to interpret because the outliers might exert strong influence on the mean and median.

#### Transcripts with high and low decay rate

We identify 200 most unstable and stable transcripts in the WT line and perform a Gene Ontology (GO) term enrichment analysis. The genes coding for unstable transcripts are disproportionally involved in development of the larval cuticle (Additional file 3: Table S1). This is consistent with the observations that numerous proteins degrade rapidly during larval development after chitin synthesis is inhibited [51]. By contrast, the stable transcripts tend to be implicated in translation (Additional file 4: Table S2), consistent with previous results [52–55].

We hypothesize that the fast decay of unstable transcripts is induced by AU-rich elements (ARE), GU-rich elements (GRE) and U-rich elements (URE), which are targeted by the RNA binding protein (RBP) [56–60]. Consistently, decay rates of mRNAs encoding these elements are statistically marginally higher than the remaining mRNAs in the WT line (Wilcoxon rank sum test, *P* = 0.09, Additional file 5: Figure S3). This pattern is much more pronounced in the KO line (*P* = 8.8 × 10^− 15^). The difference between two genetic backgrounds could be due to the fact that miR310s target three RBPs including *Rb97D, CG4119*, and *CG4896*. When miR310s are knocked out, RBPs get upregulated (Additional file 6: Table S3) and transcripts encoding elements like ARE are degraded even faster.

#### The decay rate of targets in the WT vs. KO background

**Fig. 2:**
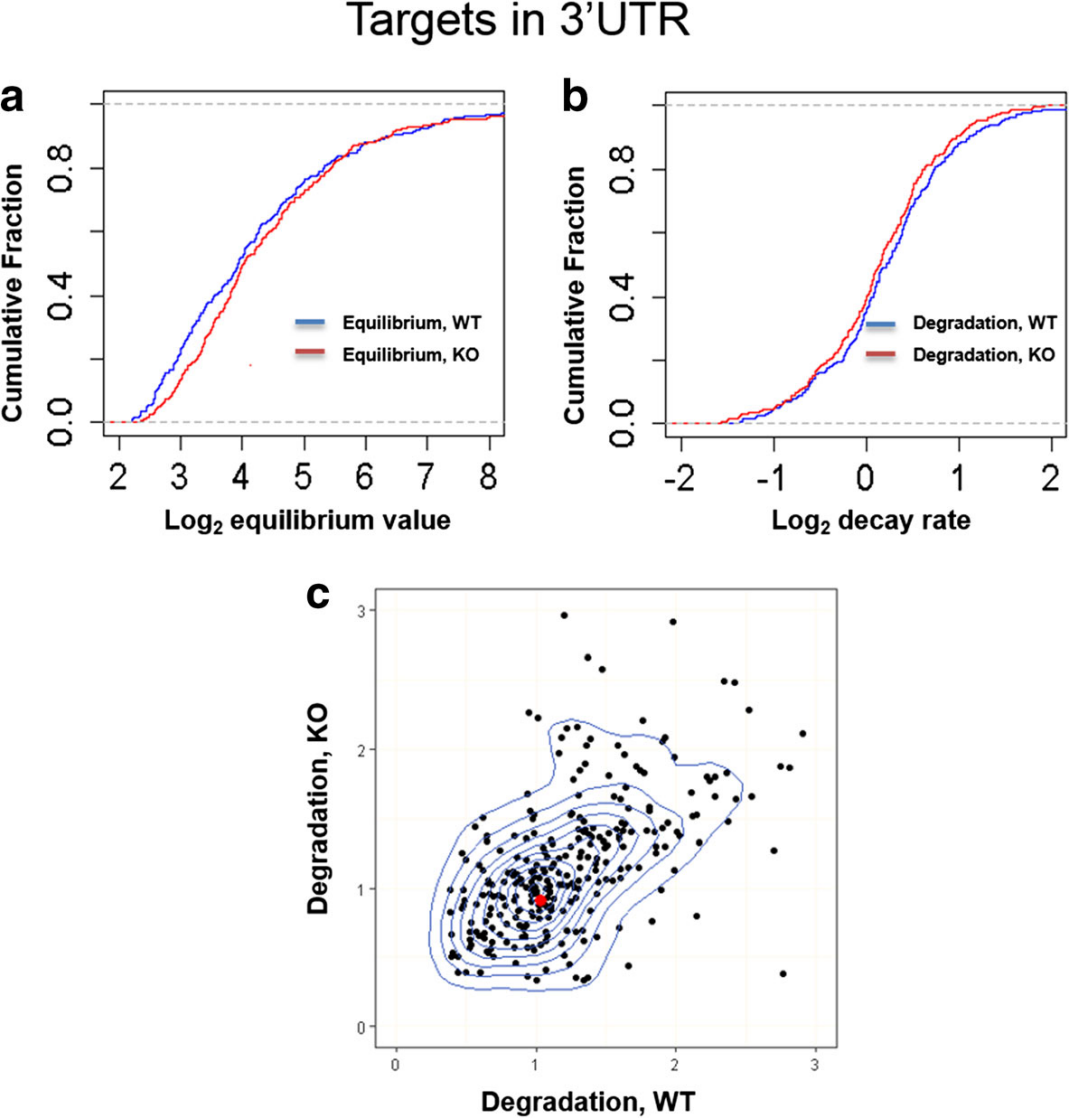
Distribution of the equilibrium expression and degradation change of 3’UTR targets between miR310s WT and KO. Targets’ degradation rates were normalized by the mode of the background decay rate. The median of equilibrium expression value is 15.3 for WT and 16.3 for KO. The degradation rate median value is 1.16 for WT and 1.11 for KO. (**a**) The cumulative distribution of equilibrium expression change. (**b**) The cumulative distribution of degradation change. (**c**) The contour distribution of miR310s targets’ degradation between WT and KO. Here, we performed a 2D kernel density estimation with an axis-aligned bivariate normal kernel, evaluated on a square grid and displaying the results with contours. The line indicates the contour estimated by the density of points. The red dot marks the densest point (1.04, 0.91). Strongly modulated target genes tend to be in the “high” or “long” group, which account for > 80% of the outliers

To quantify the contribution of miRNAs to transcript degradation (i.e., m_i_’s), we identify 292 miR310s targets (see Methods). We measure the repression effect of miR310s by the equilibrium value - X_i_ (= B_i_/D_i_) vs X_i_* (= B_i_*/d_i_) (Fig. 2a). Indeed, the distribution of X_i_* is shifted to the right suggesting higher expression of the target transcripts when the miR310s cluster is removed. The median value shows a 6.5% (=16.3/15.3–1) increase, confirming earlier suggestions of weak repression by miR310s.

We then examine cumulative distributions of D_i_ (= m_i_ + d_i_, in the WT background) vs. d_i_ (in the KO background, Fig. 2b). The distribution of D_i_ is shifted to the right, as expected. However, the shift is even smaller than in Fig. 2a. In Fig. 2b, the median values are 1.16 and 1.11, respectively. The increase contributed by m_i_ is only 4.50% (=1.16/1.11–1). Therefore, if we consider the median, the direct measurement of miRNA repression is smaller than the indirect measurement of miRNA effect based on equilibrium expression.

The analysis of the median in Fig. 2 corroborates the general view of weak repression by miRNAs. Nevertheless, the long tail of the decay distribution (Fig. 1d) makes it difficult to assess the population effect robustly. While the median is a conventional choice, we also considered the mode of the distribution as an additional inference statistic. Fig. 2c shows that the distribution of d_i_ and m_i_ + d_i_ values where the contour plot reveals the densest concentration of points centers on (1.04, 0.91). This analysis is feasible because the degradation measurements among the 292 target transcripts are clustered relatively tightly and within the same order of magnitude, and the distribution is unimodal. Thus, using the distribution mode as the statistic, the overall degradation increase contributed by miR310s may be as high as 14.3% (1.04/0.91–1). To make sure our conclusion does not depend on specific bioinformatics parameters, we re-defines miR310s targets using *D. yakuba*, a more diverged outgroup species. Our observations remain essentially unchanged (Additional file 7: Figure S4).

**Fig. 3:**
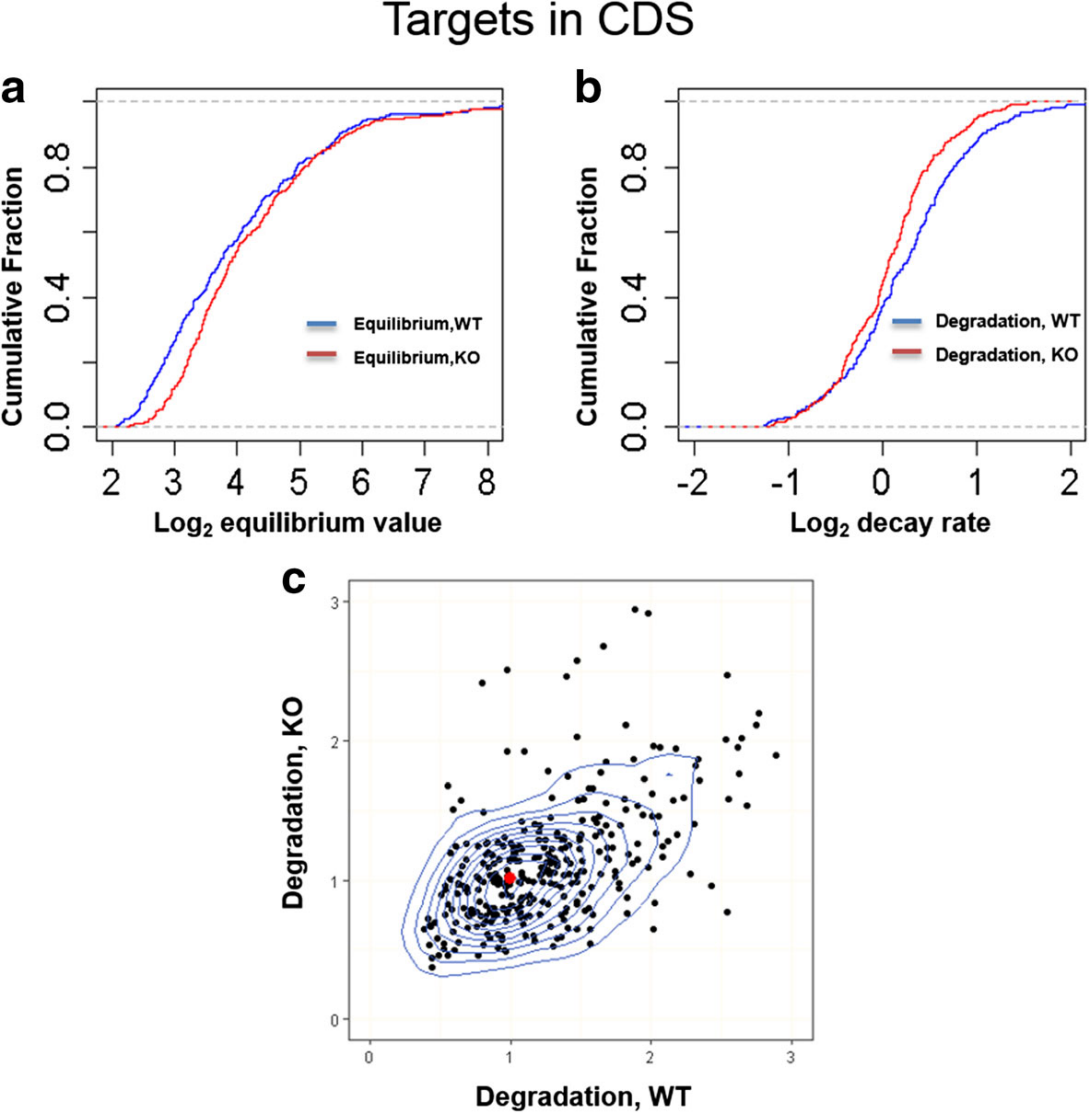
Distribution of the CDS target equilibrium expression and degradation change between miR310s WT and KO. The median degradation rate is 1.17 for WT and 1.05 for KO. (**a**) The cumulative distribution of equilibrium expression change. (**b**) The cumulative distribution of degradation change. (**c**) The contour distribution of miR310s targets’ degradation between WT and KO. The red dot on the contour plot marks the densest point (0.99, 1.01). Strongly modulated target genes tend to be in the “high” or “long” group, which account for > 80% of the outliers

We next checked if mi310s sites in coding sequences (in addition to the already considered 3’-UTRs) affect our conclusions. Such sites have been found to be functional before [61]. Analogous to the procedure for 3’-UTR targets, we extracted targets conserved in CDSs across *D. melanogaster* and *D. simulans* and examine both equilibrium and degradation change. The median equilibrium value of WT and KO is 13.0 and 14.6, respectively (Fig. 3a). That is, the increase is 12.3% (14.6/13.0–1). Correspondingly, the median degradation decrease is 11.4% (1.17/1.05–1) (Fig. 3b). However, the densest point in the contour plot shows that the degradation change is roughly − 2.0% (0.99/1.01–1) (Fig. 3c). Thus, miR310s changes degradation of CDS targets is about − 2.0% ~ 11.4%, which appears weaker than transcripts with binding sites in 3’ UTRs (4.5%~ 14.3%).

In summary, the degradation effect contributed by miR310s as measured the direct assay on transcripts with 3’-UTR binding sites is between 5 and 15% whereas the effect estimated from equilibrium expression is 6.5%. In the previous section, it was suggested that the two estimates might differ by as much as 10-fold (10 to 120%) due to the confounding factor of new transcript synthesis. Such a possibility is ruled out by this study. Given the weaker effect of targets in CDS, this possibility is even smaller for these targets.

### III. Degradation factors other than miR310s

Transcripts targeted by miR310s at their 3’ UTRs differ in the number of target sites, matching types (i.e., 8mer-1a, 7mer-1a and 7mer-m8), alternative polyadenylation (APA), whether they are also co-targeted by other miRNAs, and the length of UTRs. Since all of these factors may affect degradation [62–65] we performed a series of binary analyses to test their potential effects.

**Fig. 4:**
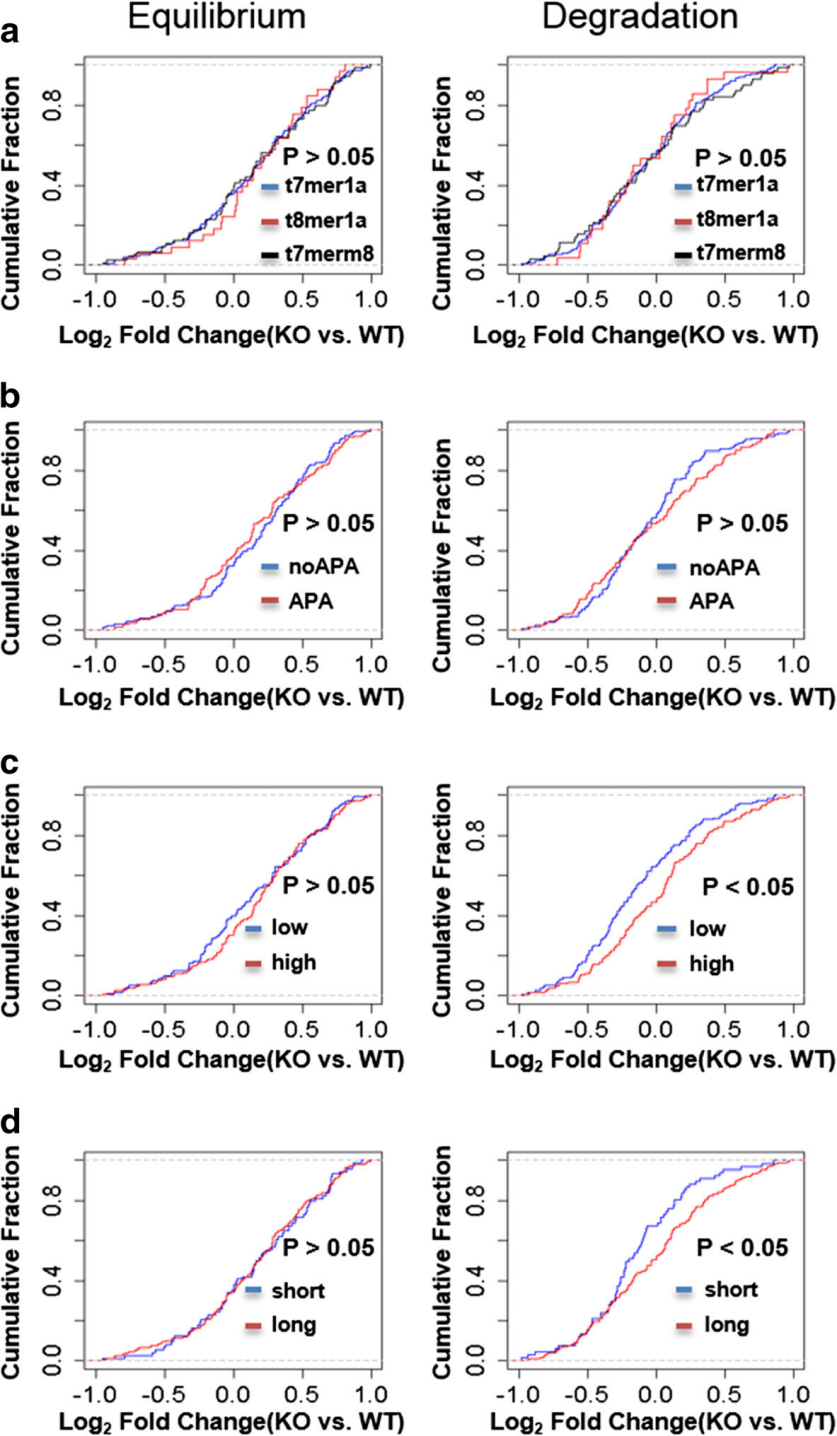
Distribution of miR310s contribution to the equilibrium and degradation of different types of miR310s 3’UTR targets. We divide the targets by (**a**) seed match types (7mer-1a, 7mer-m8, and 8mer-1a), (**b**) potential alternative polyadenylation location, (**c**) whether targeted transcripts are also targeted by other highly-expressed miRNAs or not, and (**d**) UTR length. The left panels indicate equilibrium change and the right panels show degradation change. *P* values are from the Wilcoxon rank sum test

First of all, since 90% of the targeted transcripts harbor only one miRNA site, we group them by matching types. Equilibrium expression gradually increases in the KO line according to the following order: 8mer-1a (15.1%), 7mer-1a (13.4%), and 7mer-m8 (11.9%) (Fig. 4a). Accordingly, degradation decreases by 9.4, 5.8, and 4.8%, respectively. Although the difference between groups is not significant, the trend is consistent with previous reports where 8mer-1a sites exert stronger repression intensity than 7mer-1a and 7mer-m8 [66, 67]. (We note that Figs. 2 and 4 use different statistics due to the nature of data partitioning. Figure 2 shows the difference in the median values of the control vs. KO lines whereas Fig. 4 shows the median value of the control-KO differences. The different treatments lead to the same conclusion.)

To determine whether a target can be subject to APA, we looked for the presence of the canonical polyadenylation site AATAAA or the non-canonical site ATTAAA. We classify targets into two groups: those with the APA site prior to the miR310s targeting site (the “APA” group) and those without an APA site (the “noAPA” group). Since APA results in a deletion of the miRNAs targeting site [63], we expect that the former group will show a smaller increase in expression*.* We observe the expected pattern: a 10.2% in the APA group and a 15.9% elevation in the non-APA group. Accordingly, the degradation decrease is 4.7% in APA and 6.0% in the non-APA group (Fig. 4b), although the between-group difference is not statistically significant.

Third, the miR310s cluster is only moderately expressed during the third instar larvae and one transcript could be targeted by multiple miRNAs. Based on the expression profile, we identify the top 20 most highly-expressed miRNAs. We then classify miR310s targets into two groups: “high” (transcripts co-targeted by highly-expressed miRNAs) and “low” (transcripts not targeted by highly-expressed miRNAs). Although the equilibrium changes are not significant between the “high” and “low” groups (12.0% vs. 10.1%, *P* > 0.05, Fig. 4c), the degradation changes are (− 2.7% vs. 15.2%, *P* < 0.05). Such a strong contrast suggests that during degradation, highly-expressed miRNAs exert even stronger inhibition effects on targets once miR310s are knocked out. Several possible mechanisms may account for this compensatory effect. One of them is the RNA:RNA cross talks via the sponge mechanism [68, 69]. Alternatively, the absence of one miRNA may facilitate the binding by other miRNAs. This alternative explanation is consistent with the actions of highly expressed miRNAs [45, 62, 70, 71].

Finally, we classify the targets into two groups: the subset of targets with the longest 3’ UTR among all the alternative transcripts (“long” subgroup) and another with relatively short UTR (“short” subgroup). The equilibrium changes are again not significant between these “long” and “short” groups (14.3% vs. 12.7%, *P* > 0.05, Fig. 4d), but the degradation rate changes are (2.0% vs. 12.8%, *P* < 0.05). Such a pattern suggests that the stability of an alternative transcript with longer UTR may be regulated by multiple mechanisms, with miR310s regulation representing only one layer.

In summary, the equilibrium changes are always insignificant in our tests, but the degradation changes show a significant change in two cases. Moreover, except for the “high” and “long” groups, degradation rate change is consistent with the equilibrium value change. Considering more than 50% of transcripts overlap between these two groups, the expression level may be affected by multiple factors besides degradation.

## Discussion

The pervasive weak repression of target transcripts by miRNAs has been the central conundrum about miRNA function in animals. In particular, the repressions by miRNAs are generally smaller than the level of natural variation in transcript abundance. This weak effect has led to a published view (e.g., Pinzon et al.) that the bulk of repressions by miRNAs may not be biologically relevant. Nevertheless, this view itself is also in dispute [72].

There are many hypotheses. The simplest one posits that most of these repressions are merely noise [11]. An alternative hypothesis proposes that each miRNA governs phenotypes via multiple targets in coordination [16]. Neither hypothesis is supported by experimental evidence.

An alternative solution is to question the essence of the conundrum – that target repression is generally weak. As presented in the Introduction, the claim that weak repression at the mRNA level does not reflect the strength of repression at the protein level is not compatible with available results. In fact, there are few convincing reports that support miRNAs’ roles in reducing protein output strongly [34–37].

In this study, we propose an additional possibility that has attracted relatively little attention in the literature [52]. This new hypothesis is based on the transcription-degradation coupling. For example, closely related species of yeast tend to show a strong correlation between the rate of transcription and degradation. A gene product that is more strongly degraded tends to be more highly transcribed in the same species [38]. Several of the possible molecular mechanisms of the coupling have been suggested [38–40, 73–75]. If some of these mechanisms are incorporated into the miRNA control circuitry, the indirect measurement of the equilibrium level of gene expression would under-estimate miRNA repression effect. In theory, the indirect and direct measurements could differ by orders of magnitude. This coupling effect is expressed in the S term of Eq. (2).

By turning off transcription in *Drosophila* larvae, we remove this confounding effect from estimation. Most importantly, the direct and indirect measurements of miRNA repression overlap. Although the direct measurement of transcript decay yields more variable values (both technically and biologically), it is clear that the accepted view of pervasive weak repression by miRNAs appears valid.

Given this result, we return to the original question of miRNAs’ diffuse action, i.e., weak and broad repression. Several contending hypotheses attempt to explain this phenomenon, as recently summarized by Liufu et al. (2017) and Zhao et al. (2017) [16, 72]. Some of these hypotheses suggest that weak repression is generally devoid of function [11]. In this view, only a few of the targets are truly functional where small expression differences can lead to significant phenotypic consequences [12, 15, 76]. While this view can explain what is measurable, it leaves the bulk of miRNA repression unaccounted for. Against this backdrop, the “canalization” view which posits miRNAs’ functions in stabilizing the transcriptome and, hence, canalizing the phenotypes [44, 45, 70, 77] deserves more attention. The merit of the canalization view is that it takes into account the broad actions of weak repression, since all weak repression events cumulatively contribute to the stability of the transcriptome [72]. Recently, this canalization view has been expanded from the motif structure consisting of a few nodes [71, 78, 79] to the entire RNA network [80]. This current study, by corroborating the extent of weak repression, should help to re-energize the debate.

## Conclusion

Gene regulation is expected to be direct, specific and sufficiently powerful in order to exert non-trivial phenotypic effects. Under this expectation, microRNAs (miRNAs) in metazoans are an enigmatic class of regulatory molecules. A central conundrum about miRNA function is the weak target repression. Since the new mRNA synthesis is usually not accurately accounted for, this repression effect may have been under-estimated in previous studies. We measured repression effects directly by turning off new target transcript synthesis and found that the repression effect is indeed as weak as the conventional assays suggest. Our data therefore rekindle the debate on the diffusive actions of miRNAs.

## Methods

### Fly culture and the miR310s knock-out stock

Fly larvae were reared at 25 °C and were fed a normal diet of corn and soybean meal, agar, and molasses supplemented with yeast. miR310s were knocked out by P-element transposition excision and maintained as a stock in the lab [81].

### ActD treatment

We applied ActD (Sigma) that was soluble in the Phosphate Buffer Saline (PBS) at a raw concentration of 0.5 mg/ml. To get a suitable ActD concentration, different doses of ActD were tried and 80 μg/ml ActD was applied to sufficiently inhibit synthesis. L3 larvae were soaked in 800 μl of 80 μg/ml ActD in a 35 mm cell culture plate, with the solution just covering larval bodies. The larvae could breath under this volume of water but could not climb out of the liquid. RNA-Seq samples from WT and KO lines were collected at 0, 2, 4, and 8 h with two biological replicates. High correlation of all mRNAs and targets were observed between the two biological replicates (Additional file 1: Figure S1).

### Reverse transcription – Quantitative real-time polymerase chain reaction (qRT-PCR)

The isolated RNA was reverse-transcribed into cDNA using the PrimeScript II 1st Strand cDNA Synthesis Kit (TaKaRa). cDNA was amplified using the primer sets listed in the Additional file 8: Table S3. RP49 was used as an internal control. The SYBR Premix Ex Taq (TaKaRa) was used in accordance with the manufacturer’s instructions. Quantitative real-time reverse transcription PCR analysis was performed using an applied biosystems 7900HT Real Time System (ThermoFisher Scientific). Decay rates estimated by qPCR were highly correlated with those derived from RNA-seq (Additional file 8: Figure S5). Primers are listed in Additional file 9: Table S4.

### Library preparation and RNA-Seq

Total RNAs were extracted using the TRIzol Reagent (Ambion). RNA quality was assessed using 1% agarose gel electrophoresis. Five microgram of total RNA was used and polyA positive RNAs were isolated using the Dynabeads mRNA DIRECT Kit (Invitrogen). RNA-seq libraries were prepared according to the standard Illumina RNA-seq Library preparation protocol. Libraries were barcoded, pooled, and sequenced using Illumina HiSeq V4 with 125-bp paired reads. The raw sequence data were deposited in the Genome Sequence Archive [82] of the BIG Data Center [83], which is maintained by the Beijing Institute of Genomics (BIG) of the Chinese Academy of Sciences. The website is http://bigd.big.ac.cn/gsa and the accession number is PRJCA000381.

### RNA-seq data analysis

The gene annotation file was downloaded from the Ensembl database. RNA-seq reads were mapped to *D. melanogaster* Reference Genome (BDGP6.83) using tophat [84] and transcripts’ expression value was quantified as “Fragments Per Kilobase of exon per Million fragments mapped” (FPKM) using Cufflinks [85].

### Identification of targets

It has been illustrated that the conservation of miRNA’s target site sequences at 3′ untranslated regions (3’UTR) indicates functionality [86] and miRNA’s target sites at coding sequences (CDS) are also conserved and functional between species [61]. Since miR310s are relatively young (predating the *D. melanogaster* and *D. pseudoobscura*), we identified targets based on the conservation of seed sequence between *D. melanogaster* and two relatively closely related species: *D. simulans* and *D. yakuba*. miR310s CDS targets are conserved between *D.melanogaster* and *D.simulans*. Transcripts targeted by miR310s were identified using the TargetScan algorithm [7, 47, 87, 88]. 3’UTR annotation was extracted from BDGP6.83. The whole genome alignments were dm6, droSim1, and droYak3 available at the UCSC Genome Browser. miRNA expression during the L3 larval stage was ranked according to previous studies [47, 48].

### Decay rate calculation

Although the lifetime of rRNA is measured in days [89] and the expression samples are collected at relatively early time points, normalization is indispensable between different time points. After we used FPKM to normalize for gene length and sequencing depth and chose 10 most stable ribosomal protein mRNAs that were relatively and increasingly expressed at later time points to perform normalization across samples. Due to experimental variance, long half-lives computed for stable transcripts were often inaccurate. Therefore, mRNAs with half-lives > 15 h were filtered out before further analysis. Moreover, after normalization, a subset of transcripts’ expression became very low and could lead to misleading inference of D. Therefore, transcripts with FPKM lower than 5 in any one sample were also filtered out. In eq. (5) and (6), normalized FPKM were used for linear fitting. D was estimated as the regression slope. Since the range of D is small (0–0.4, Fig. 1d), the mode (the most frequent value, 0.12 in WT and 0.14 in KO), in addition to the median, can be used to estimate the background decay rate pattern. To enable direct comparisons, we divided each individual value by the mode (0.12 in WT, 0.14 in KO) in each genetic background separately. All calculations were conducted in R [90].

### Gene ontology analysis

The top 200 most stable and unstable transcripts were extracted to examine their Gene Ontologies. GO analysis enrichment was performed by GO:TermFinder with the 0.05 false discovery rate cutoff [91, 92].

### mRNAs with ARE, GRE and URE motifs analysis

ARE, GRE and URE motifs refer to the sequence, ATTTA, AWTAAA, GTTTG, TTTGTTT, WTTTW, WWTTTWW, WWWTTTWWW, WWWWTTTWWWW, and WWWWWTTTWWWWW (W is A/U). Genes were extracted from the ARE2 website (http://nibiru.tbi.univie.ac.at/AREsite2/genes) [93]. Genes with the above listed motifs in 5’UTR, Exon, Intron, CDS and 3’UTR simultaneously were considered as potentially unstable mRNAs. We found about 490 transcripts fitting the criteria.

## Additional files

Additional file 1: **Figure S1.** Correlation of all transcript and target expression (log_2_ transformed) between two biological replicates at 0 h, 2 h, 4 h, and 8 h. (a-d) results from the WT line, (e-h) results from the KO line. (TIF 368 kb)

Additional file 2: **Figure S2.** Half-lives of whole transcripts in *D. melanogaster* third instar larvae. (TIF 157 kb)

Additional file 3: **Table S1.** Relating mRNA stability to gene function using a Gene Ontology (GO) analysis in the WT line (the 200 least stable genes). (PDF 133 kb)

Additional file 4: **Table S2.** Relating mRNA stability to gene function using a Gene Ontology (GO) analysis in the WT line (the 200 most stable genes). (PDF 163 kb)

Additional file 5: **Figure S3.** Cumulative distribution of mRNA decay rates of with or without AU-rich, GU-rich and U-rich elements in 3’UTR, 5’UTR, Intron, Exon, and CDS simultaneously in (a) the WT and (b) KO line. AU-rich, GU-rich and U-rich element: ATTTA, AWTAAA, GTTTG, TTTGTTT, WTTTW, WWTTTWW, WWWTTTWWW, WWWWTTTWWWW, and WWWWWTTTWWWWW. W: A/U. *P* values are from Wilcoxon rank sum tests. (TIF 98 kb)

Additional file 6: **Table S3.** The decay rates of three targets that belong to the RNA binding protein (RBP) family. (PDF 196 kb)

Additional file 7: **Figure S4.** (a-c) Distribution of the equilibrium expression and degradation change of 3’UTR targets conserved between *D. melanogaster* and *D. yakuba*. Targets’ degradation rates were normalized by the mode of the background decay rate. The median of equilibrium expression value is 15.3 for WT and 16.4 for KO. The median of degradation rate is 1.19 for WT and 1.14 for KO. (a) The cumulative distribution of equilibrium expression change. (b) The cumulative distribution of degradation change. (c) The contour distribution of miR310s targets’ degradation between WT and KO. The red dot on the contour plot marks the densest point (1.04, 0.92). (TIF 212 kb)

Additional file 8: **Figure S5.** The relationship between the decay rate calculated by qRT-PCR and mRNA-seq. RP49 is used as the reference. (TIF 72 kb)

Additional file 9: **Table S4.** Real-time polymerase chain reaction primers used for mRNA quantification. (PDF 198 kb)

## Abbreviations

*: The state for KO
3’UTR: 3′ untranslated regions
ActD: Actinomycin D
*a*_*ij*_: Regulation strength of gene *j* on gene *i*
ARE: AU-rich elements
b: Constant basal transcription rate
B: Synthesis rate
d: Degradation rate for KO
D: Degradation rate for WT
*D. melanogaster*: *Drosophila melanogaster*
*D. pseudoobscura*: *Drosophila pseudoobscura*
*D. simulans*: *Drosophila simulans*
*D. yakuba*: *Drosophila yakuba*
Eqs: Equation
FPKM: Fragments Per Kilobase of exon per Million fragments mapped
GRE: GU-rich elements
GRN: Gene regulatory network
KO: Knockout
L3: The third instar larvae
miR310s: MiR310 cluster
miRNAs: MicroRNAs
mRNA: Message RNA
PBS: Phosphate Buffer Saline
RNA-seq: mRNA high-throughput sequencing
rRNA: Ribosomal RNA
S: Other genes’ effect on gene i
TFs: Transcription factors
UCSC: University of California Santa Cruz
URE: U-rich elements
WT: Wild-type
X: Equilibrium state
x_t_: mRNA concentration of a transcript at time t
