# Supplementary figures and images for: Direct measurement of pervasive weak repression by microRNAs and their role at the network level
Source: BMC Genomics. 2018 May 15;19:362. doi: 10.1186/s12864-018-4757-z (PMC5952853; doi:10.1186/s12864-018-4757-z)

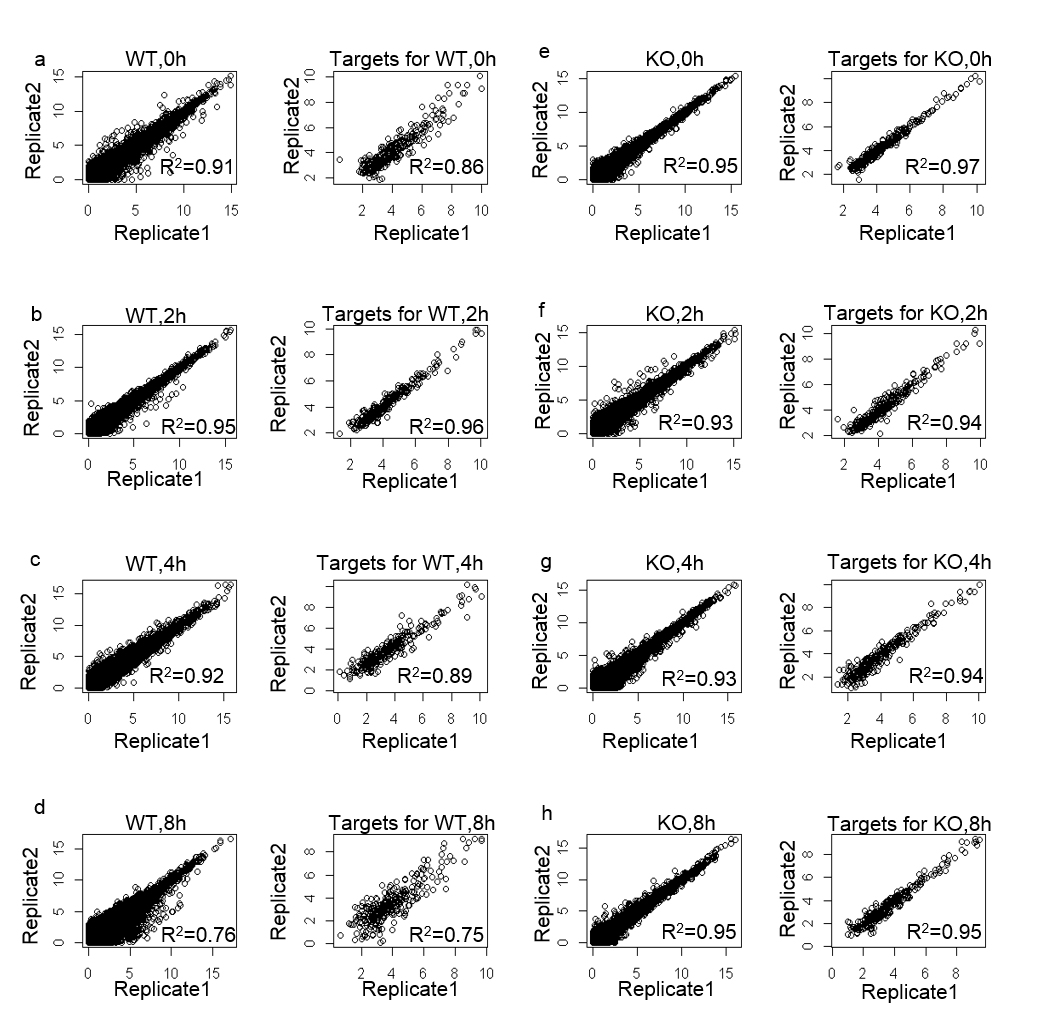

Supplement: Supplementary file 1 — Figure S1. Correlation of all transcript and target expression (log2 transformed) between two biological replicates at 0 h, 2 h, 4 h, and 8 h. (a-d) results from the WT line, (e-h) results from the KO line. (TIF 368 kb) [file 12864_2018_4757_MOESM1_ESM.tif]

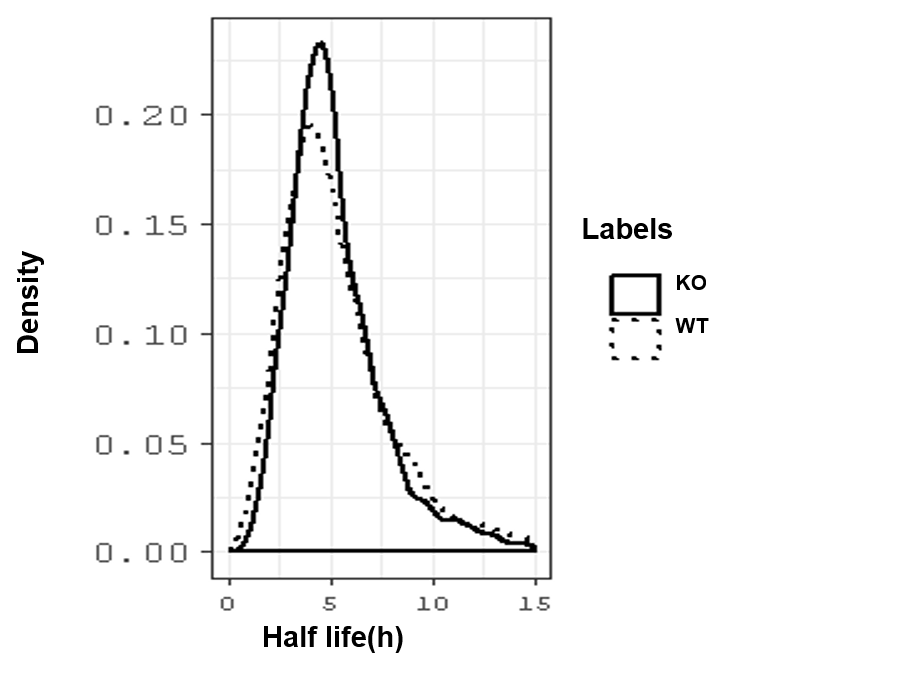

Supplement: Supplementary file 2 — Figure S2. Half-lives of whole transcripts in D. melanogaster third instar larvae. (TIF 157 kb) [file 12864_2018_4757_MOESM2_ESM.tif]

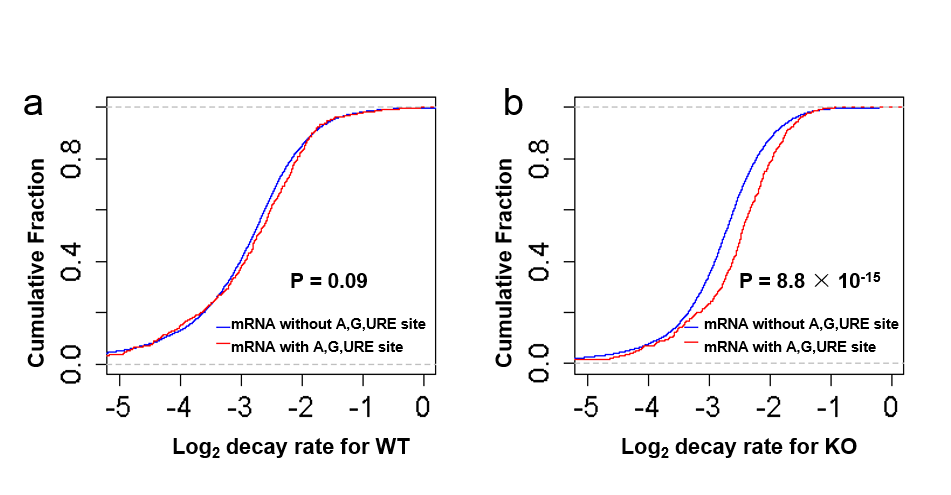

Supplement: Supplementary file 5 — Figure S3. Cumulative distribution of mRNA decay rates of with or without AU-rich, GU-rich and U-rich elements in 3’UTR, 5’UTR, Intron, Exon, and CDS simultaneously in (a) the WT and (b) KO line. AU-rich, GU-rich and U-rich element: ATTTA, AWTAAA, GTTTG, TTTGTTT, WTTTW, WWTTTWW, WWWTTTWWW, WWWWTTTWWWW, and WWWWWTTTWWWWW. W: A/U. P values are from Wilcoxon rank sum tests. (TIF 98 kb) [file 12864_2018_4757_MOESM5_ESM.tif]

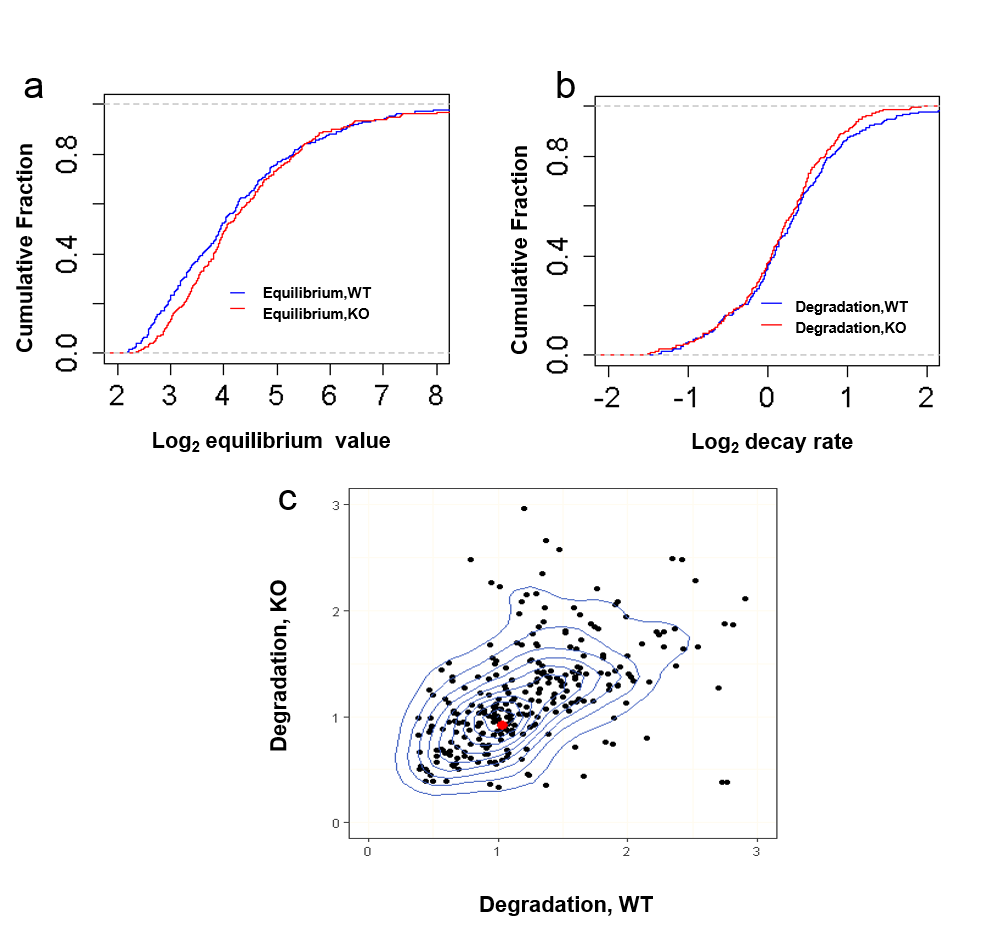

Supplement: Supplementary file 7 — Figure S4. (a-c) Distribution of the equilibrium expression and degradation change of 3’UTR targets conserved between D. melanogaster and D. yakuba. Targets’ degradation rates were normalized by the mode of the background decay rate. The median of equilibrium expression value is 15.3 for WT and 16.4 for KO. The median of degradation rate is 1.19 for WT and 1.14 for KO. (a) The cumulative distribution of equilibrium expression change. (b) The cumulative distribution of degradation change. (c) The contour distribution of miR310s targets’ degradation between WT and KO. The red dot on the contour plot marks the densest point (1.04, 0.92). (TIF 212 kb) [file 12864_2018_4757_MOESM7_ESM.tif]

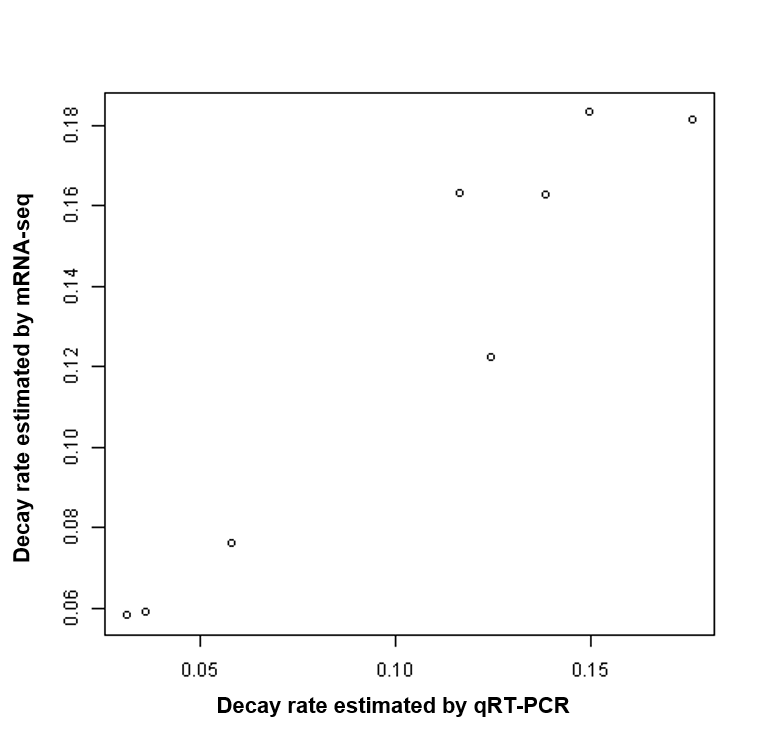

Supplement: Supplementary file 8 — Figure S5. The relationship between the decay rate calculated by qRT-PCR and mRNA-seq. RP49 is used as the reference. (TIF 72 kb) [file 12864_2018_4757_MOESM8_ESM.tif]
