# Supplementary material for: Direct measurement of pervasive weak repression by microRNAs and their role at the network level
Source: BMC Genomics. 2018 May 15;19:362. doi: 10.1186/s12864-018-4757-z (PMC5952853; doi:10.1186/s12864-018-4757-z)
Supplement: Supplementary file 3 — Table S1. Relating mRNA stability to gene function using a Gene Ontology (GO) analysis in the WT line (the 200 least stable genes). (PDF 133 kb) [file 12864_2018_4757_MOESM3_ESM.pdf]

Table S1. Relating mRNA stability to gene function using a Gene Ontology (GO) analysis in the WT line (the 200 least stable genes)

GO term		
Biological process	P value	FDR
chitin-based cuticle development	2.34E-12	1.74E-08
cuticle development	6.25E-10	2.32E-06
cellular macromolecule metabolic process	3.85E-07	9.51E-04
Molecular function		
structural constituent of chitin-based larval cuticle	1.97E-13	5.20E-10
structural constituent of chitin-based cuticle	7.02E-13	9.25E-10
structural constituent of cuticle	1.55E-12	1.36E-09
structural molecule activity	2.11E-05	1.39E-02
Cellular component		
extracellular matrix	6.61E-11	8.36E-08
extracellular region part	2.39E-05	4.31E-03
extracellular region	8.56E-07	3.61E-04
membrane-bounded organelle	1.30E-04	1.83E-02
organelle	8.58E-05	1.36E-02
intracellular organelle part	3.29E-06	8.33E-04
intracellular part	4.17E-04	4.79E-02
intracellular organelle	1.56E-04	1.98E-02
organelle part	1.63E-05	3.45E-03
protein complex	2.00E-06	6.33E-04
macromolecular complex	8.50E-09	5.37E-06

Significant GO terms and relevant P values and FDR (False Discovery rate)are shown. FDR cutoff = 0.05.
