# Supplementary material for: Direct measurement of pervasive weak repression by microRNAs and their role at the network level
Source: BMC Genomics. 2018 May 15;19:362. doi: 10.1186/s12864-018-4757-z (PMC5952853; doi:10.1186/s12864-018-4757-z)
Supplement: Supplementary file 4 — Table S2. Relating mRNA stability to gene function using a Gene Ontology (GO) analysis in the WT line (the 200 most stable genes). (PDF 163 kb) [file 12864_2018_4757_MOESM4_ESM.pdf]

Table S2. Relating mRNA stability to gene function using a Gene Ontology (GO) analysis in the WT line (the 200 most stable genes)

GO term	P value	FDR
Biological process	P value	FDR
cytoplasmic translation	3.71E-14	2.75E-10
translation	2.57E-11	9.55E-08
cellular protein metabolic process	1.44E-06	9.72E-04
protein metabolic process	9.18E-05	3.58E-02
cellular macromolecule metabolic process	4.99E-06	2.64E-03
cellular process	3.60E-05	1.57E-02
cellular macromolecule biosynthetic process	5.42E-07	4.46E-04
macromolecule biosynthetic process	9.42E-07	6.99E-04
organic substance biosynthetic process	1.18E-05	5.49E-03
biosynthetic process	8.67E-06	4.29E-03
cellular biosynthetic process	4.37E-06	2.49E-03
peptide biosynthetic process	3.53E-11	8.73E-08
peptide metabolic process	1.24E-10	1.85E-07
cellular amide metabolic process	8.80E-10	1.09E-06
cellular nitrogen compound metabolic process	2.53E-06	1.56E-03
organonitrogen compound biosynthetic process	5.27E-08	5.59E-05
amide biosynthetic process	8.29E-11	1.54E-07
cellular nitrogen compound biosynthetic process	5.75E-08	5.33E-05
gene expression	3.87E-05	1.60E-02
ribosome assembly	1.27E-04	4.72E-02
Molecular function complete		
structural constituent of ribosome	4.83E-11	1.27E-07
structural molecule activity	7.85E-06	6.89E-03
RNA binding	7.35E-07	9.68E-04
Cellular component complete		
intracellular ferritin complex	1.00E-03	5.06E-02
Continued		
intracellular part	1.03E-04	6.84E-03
intracellular	1.06E-04	6.70E-03
cell part	3.23E-04	1.86E-02
cell	3.23E-04	1.94E-02
ferritin complex	1.00E-03	4.86E-02
protein complex	8.68E-04	4.58E-02
macromolecular complex	7.83E-07	9.01E-05
cytosolic large ribosomal subunit	7.55E-12	3.18E-09
cytosolic part	5.72E-12	3.62E-09
cytosol	3.24E-08	5.12E-06
cytoplasmic part	5.08E-09	1.07E-06
cytoplasm	3.55E-05	2.99E-03
large ribosomal subunit	6.64E-09	1.20E-06
ribosomal subunit	9.63E-11	2.44E-08
intracellular organelle part	2.96E-05	2.67E-03
intracellular organelle	4.92E-05	3.89E-03
organelle	5.21E-05	3.87E-03
organelle part	7.24E-05	5.09E-03
intracellular ribonucleoprotein complex	1.46E-06	1.42E-04
ribonucleoprotein complex	1.46E-06	1.54E-04
ribosome	2.83E-11	8.96E-09
intracellular non-membrane-bounded organelle	4.53E-07	6.37E-05
non-membrane-bounded organelle	4.53E-07	5.74E-05
cytosolic ribosome	2.40E-14	3.03E-11
cytosolic small ribosomal subunit	3.90E-04	2.15E-02

Significant GO terms and relevant P values and FDR (False Discovery rate) are shown. FDR cutoff = 0.05.
