# Supplementary material for: Direct measurement of pervasive weak repression by microRNAs and their role at the network level
Source: BMC Genomics. 2018 May 15;19:362. doi: 10.1186/s12864-018-4757-z (PMC5952853; doi:10.1186/s12864-018-4757-z)
Supplement: Supplementary file 6 — Table S3. The decay rates of three targets that belong to the RNA binding protein (RBP) family. (PDF 196 kb) [file 12864_2018_4757_MOESM6_ESM.pdf]

Table S3. The decay rates of three targets that belong to the RNA binding protein (RBP) family

Targeting genes	Targeting transcripts	Decay rates for WT	Decay rates for KO
Rb97D (CG6354)	FBtr0100340	0.15	0.13
CG4119	FBtr0070799	0.23	0.18
CG4896	FBtr0335394	0.24	0.23
