# Supplementary material for: Direct measurement of pervasive weak repression by microRNAs and their role at the network level
Source: BMC Genomics. 2018 May 15;19:362. doi: 10.1186/s12864-018-4757-z (PMC5952853; doi:10.1186/s12864-018-4757-z)
Supplement: Supplementary file 9 — Table S4. Real-time polymerase chain reaction primers used for mRNA quantification. (PDF 198 kb) [file 12864_2018_4757_MOESM9_ESM.pdf]

Table S4. Real-time polymerase chain reaction primers used for mRNA quantification.

Gene	Forward primer	Reverse primer
Jra	AAGCAAAGTTCCCACCCACT	GGATTCGGGGTGGATGTGTT
Mef2	CAAGCTGTACCAGTACGCCA	GTTCGCGGAGTGAGTGTGTA
Mad	GACGAAGAGGAGAAGTGGGC	GGGAGACCTGTAATCGTCCG
Ps	ACGGGTGCTAGGGTCAAGAT	CGACCATGATAGCCTCCGTG
Twin	TGCCCACATCCGCATATACC	TGGCTAGCCGTATGCATCAG
Nop60B	AAGACTGGCTTCATCAACCTG	GCAACCCGTGACTTTGGGA
Notch	CGCTTCCTGCACAAGTGTC	GCGCAGTAGGTTTTGCCATT
Sqd	GGTCGATGTTAAGCGTGCGA	GTCCGTCCCACTGGTTGTT
RP49	ATGCTAAGCTGTCGCACAAA	GTTCGATCCGTAACCGATGT
